# Amenable Mortality in Children under 5: An Indicator for Identifying Inequalities in Healthcare Delivery: A Review

**DOI:** 10.3390/children11070764

**Published:** 2024-06-24

**Authors:** Eduardo Navarro-Jimenez, Pedro Saturno-Hernández, Marta Jaramillo-Mejía, Vicente Javier Clemente-Suárez

**Affiliations:** 1 Facultad de Ciencias de la Salud, Universidad Simón Bolívar, Barranquilla 080005, Colombia; eduardo.navarro@unisimon.edu.co; 2 National Institute of Public Health (INSP), Cuernavaca 62100, Mexico; pedro.saturno@insp.mx; 3 Facultad de Ciencias de la Salud, Departamento de Salud Pública y Medicina Comunitaria, Universidad Icesi, Cali 760031, Colombia; mcjara@icesi.edu.co; 4 Faculty of Sports Sciences, Universidad Europea de Madrid, Tajo Street, s/n, 28670 Madrid, Spain; 5 Grupo de Investigación en Cultura, Educación y Sociedad, Universidad de la Costa, Barranquilla 080002, Colombia

**Keywords:** amenable (treatable) mortality, under-5 mortality, timely medical care, healthcare service quality, universal health coverage, respiratory disease, perinatal period

## Abstract

Universal health coverage has been proposed as a strategy to improve health in low- and middle-income countries, but this depends on a good provision of health services. Under-5 mortality (U5M) reflects the quality of health services, and its reduction has been a milestone in modern society, reducing global mortality rates by more than two-thirds between 1990 and 2020. However, despite these impressive achievements, they are still insufficient, and most deaths in children under 5 can be prevented with the provision of timely and high-quality health services. The aim of this paper is to conduct a literature review on amenable (treatable) mortality in children under 5. This indicator is based on the concept that deaths from certain causes should not occur in the presence of timely and effective medical care. A systematic and exhaustive review of available literature on amenable mortality in children under 5 was conducted using MEDLINE/PubMed, Cochrane CENTRAL, OVID medline, Scielo, Epistemonikos, ScienceDirect, and Google Scholar in both English and Spanish. Both primary sources, such as scientific articles, and secondary sources, such as bibliographic indices, websites, and databases, were used. Results: The main cause of amenable mortality in children under 5 was respiratory disease, and the highest proportion of deaths occurred in the perinatal period. Approximately 65% of avoidable deaths in children under 5 were due to amenable mortality, that is, due to insufficient quality in the provision of health services. Most deaths in all countries and around the world are preventable, primarily through effective and timely access to healthcare (amenable mortality) and the management of public health programs focused on mothers and children (preventable mortality).

## 1. Introduction

Health is a fundamental human right, and Universal Health Coverage (UHC) is recognized as a critical means to achieve that right [1]. The objective of UHC is expressed in the United Nations’ 2030 Agenda as part of the Sustainable Development Goals (SDGs) under Goal 3, which focuses on health (target 3.8) [2].

Globally, under-5 mortality has decreased by over two-thirds between 1990 and 2020, dropping from 93 to 39 deaths per 1000 live births [3]. Indeed, mortality rates have declined across all age groups over the last five decades, with the greatest reductions observed in children under five [4,5]. Despite these impressive advancements, inequalities persist among countries and within them (regional disparities), especially in low- and middle-income countries. Furthermore, more than half of these early childhood deaths were due to conditions that could have been prevented or treated with simple and affordable interventions, making them amenable [6,7,8].

Although infant mortality rates have long been used in global assessments of a country’s or region’s health services, Rutstein et al. (1976) proposed a more comprehensive and systematic approach, including other causes of mortality, to assess the quality of care as an outcome measure [9]. A 5% reduction in GDP per capita in 2020 was estimated to cause an additional 282,996 deaths in children under 5. At 10% and 15%, recessions led to higher losses of under-5 lives, increasing to 585,802 and 911,026 additional deaths, respectively [10].

Amenable (treatable) mortality is a subset of the broader concept of avoidable mortality, which includes deaths that can be prevented (reduction in incidence) and those that can be treated (reduction in case fatality) [11]. While healthcare can influence the former, its greatest impact lies in the latter, i.e., amenable mortality [12]. In this sense, amenable mortality is understood as an indicator of the quality and accessibility of healthcare systems, relating to deaths that can be prevented through timely and high-quality medical diagnosis and treatment, whereas preventable mortality encompasses deaths that could be avoided through public health interventions such as health promotion, disease prevention, specific protection measures, and the implementation of sectoral and cross-sectoral public policies [13,14]. Therefore, avoidable mortality encompasses both amenable and preventable mortality [15,16]. In the same vein, the World Health Organization (WHO) outlines the difference between potentially avoidable premature mortality, potentially preventable mortality, and potentially treatable mortality [17,18].

The purpose of this study was to conduct a narrative review and analyze the trends in amenable mortality rates among children under five in countries and subregions, their causes according to ICD-10 where possible, and assess potential inequalities in healthcare delivery influencing amenable mortality in children.

## 2. Materials and Methods

Amenable (treatable) mortality is defined as deaths that can be prevented through timely and effective healthcare interventions. This review focused specifically on amenable mortality among children under five years of age, a critical public health concern.

### 2.1. Search Strategy

A comprehensive search was conducted in the following electronic databases: PubMed, Embase, ScienceDirect, Scopus, Web of Science, and Epistemonikos. The search terms used were:

- “amenable mortality”
- “treatable mortality”
- “child”

These terms were combined and searched in both English and Spanish to ensure inclusivity and comprehensiveness.

### 2.2. Inclusion and Exclusion Criteria

*Inclusion Criteria:*
  1. Studies investigating amenable mortality in children under five years old.
  2. Articles published in English, Spanish, or Portuguese.
  3. Full-text articles available.
*Exclusion Criteria:*
  1. Studies not focusing on amenable mortality (e.g., studies focusing solely on preventable mortality or all-cause mortality).
  2. Reviews, editorials, letters, or opinion pieces.
  3. Articles without full-text availability.

### 2.3. Data Extraction

A standardized data extraction form was utilized to collect relevant information from the included studies. The extracted data included:

- Study characteristics: Authors, publication year, and study design.
- Population characteristics: Age range and sample size.
- Methods: Data sources, inclusion/exclusion criteria.
- Outcomes: Amenable mortality rates and causes of death.

### 2.4. Data Synthesis

A narrative synthesis of the extracted data was conducted. This synthesis involved summarizing the findings of the included studies, identifying key themes and patterns, and discussing the overall body of evidence on amenable mortality in children under five years of age.

### 2.5. Study Selection

The initial database search identified a total of 1765 articles from various sources:

- PubMed (*n* = 383)
- Epistemonikos (*n* = 27)
- ScienceDirect (*n* = 646)
- SciELO (*n* = 27)
- Scopus (*n* = 460)
- Web of Science (*n* = 217)

**Figure 1:**
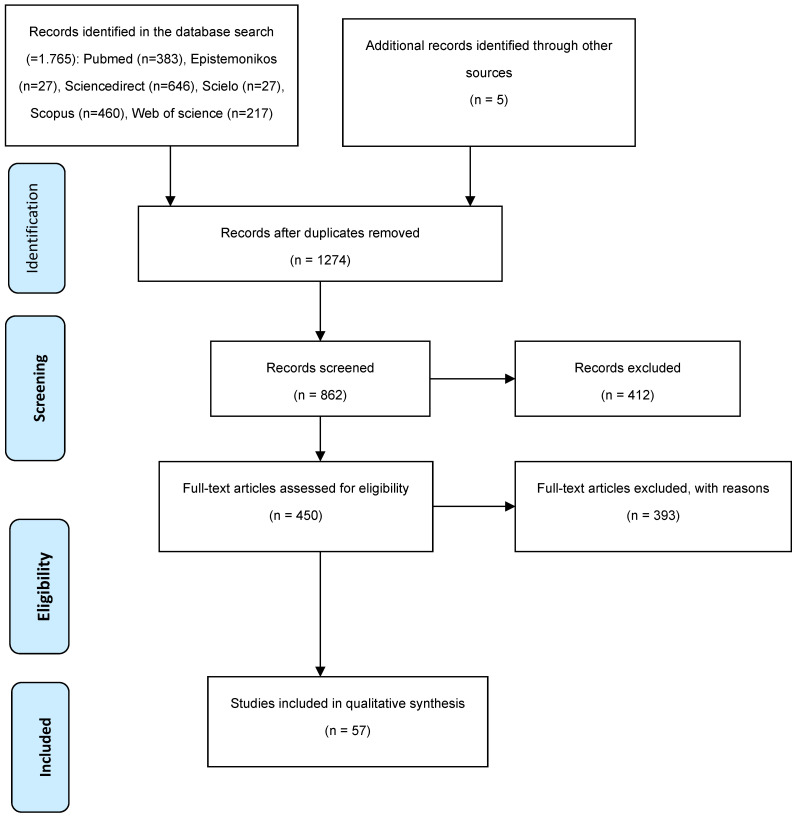
PRISMA flow diagram for study selection.

After removing duplicates, 1274 articles remained. Following a full-text review, 57 articles were included in the analysis. The selection process is illustrated in the PRISMA flow diagram (Figure 1).

### 2.6. Results Overview

The search yielded 1234 unique records. After applying the inclusion and exclusion criteria, 45 studies were deemed eligible for full-text review. Of these, 21 studies met the final inclusion criteria and were included in the qualitative synthesis.

## 3. Results

The 57 identified articles related to treatable mortality in children under 5 years old provide valuable insights. Among these articles: 31 focus on countries with low to middle incomes, 19 originate from high-income countries and 7 studies are based on global data (Table 1).

### 3.1. Low and Middle-Income Countries (LMICs)

Using data from the World Bank, Truche et al. conducted a study on the association between surgical workforce and potentially avoidable infant mortality. They found a statistically significant association between the number of healthcare professionals and a lower mortality rate in children under 5 years of age. Moreover, they observed that increasing the surgical workforce to 40 professionals per 100,000, as recommended by the Lancet Commission on Global Surgery, could prevent approximately 270,000 to 600,000 potentially avoidable deaths in children under 5 years. Most of the prevented deaths (61%) were in neonates [22,23]. These findings have significant implications for policymakers and healthcare providers in low and middle-income countries, suggesting that increasing the number of surgical workers could be an effective way to reduce infant mortality. The study also highlights the need for further research on the impact of surgical care on infant mortality, which could help identify the most effective ways to increase access to surgical care in low and middle-income countries.

In a study analyzing aggregated data from 2000 to 2014 in Latin American and Caribbean countries, Álvarez, Aburto, and Canudas-Romo found that treatable diseases contribute the most to the variability gap in life expectancy in both infant and childhood mortality (from 0 to 5 years) [24].

Aburto et al. demonstrated a significant reduction in overall mortality in male children but revealed inequality with female children’s mortality from 1990 to 2015 across all 32 Mexican states. The improvements were largely attributed to reductions in infectious and respiratory diseases, which are among the most treatable causes of mortality. However, substantial disparities persist between states with low and high economic development, indicating significant room for improvement. The study recommends that the Mexican government continue investing in programs to enhance children’s health, such as the Seguro Popular program, while addressing income inequality and improving access to healthcare in states with low economic development. Furthermore, addressing social determinants of health, such as poverty, malnutrition, and lack of education, especially among mothers of children under 5 years, is essential [25].

In Brazil, the Estratégia de Saúde da Família (ESF; Family Health Strategy), a community-based primary care program that started in 1994, has generated a beneficial impact on mortality rates. It has shown reductions in amenable mortality, perinatal mortality, infectious and circulatory diseases, as well as infant mortality rates, consistent with the implementation of ESF and the percentage of coverage in different Brazilian states. The success of ESF in reducing mortality rates in Brazil can be attributed to several reasons. Firstly, ESF provides comprehensive primary care services to families in their homes and communities, ensuring access to preventive care and treatment of chronic diseases. Secondly, ESF operates with a healthcare team comprising doctors, nurses, and community health workers, enabling a more comprehensive and coordinated approach to healthcare. Thirdly, ESF focuses on prevention and early intervention, which contributes to reducing the severity of illnesses and the risk of death [26,27]. The effect of ESF on unattended deaths was slightly stronger in municipalities with a higher human development index, indicating inequalities in healthcare provision in rural areas [28].

Another study found that expanding ESF coverage was associated with a reduction in mortality from amenable outpatient conditions, such as respiratory diseases (COPD and asthma), epilepsy, and gastric ulcers. Asthma mortality in childhood and adolescence in Brazil showed a decline in all age groups evaluated, except in the 5 to 9-year-old age group, with a more significant reduction observed in children under 5 during the 20-year study period [29]. The decrease in asthma-related mortality was observed in all geographic regions of the country. However, higher mortality rates were found in the Northeast and North regions, possibly reflecting socioeconomic inequities that result in difficulties in accessing and limited quality of healthcare resources. There was a higher vulnerability among children under 5, underscoring the need for increased attention and prioritization of health actions [30].

In a study analyzing regional mortality patterns due to perinatal causes in Brazilian children, it was found that these represent 57% of all infant deaths, with congenital malformations accounting for 11.2% of these deaths. Mortality levels were higher in the Northeast and North regions and lower in the South and Southeast regions, with the Central-West region falling in between. The study highlighted the need for interventions to reduce perinatal mortality rates in Brazil, such as improving access to healthcare, nutrition, and education in rural areas, reducing extreme poverty and teenage pregnancy [31].

In an ecological study at a public hospital in Brazil, De Assis et al. found that 41.4% of deaths were classified as difficult to reduce, 28.3% as reducible, and 30.4% as avoidable [32].

In Kenya, most women would be willing to give birth in a healthcare facility if it were affordable and accessible to them. However, various barriers prevent this from happening. These barriers include cost, distance to healthcare facilities, cultural beliefs, and lack of information [33]. The study suggests that promoting health for women and their partners can help them understand the importance of prenatal care and plan ahead for childbirth with qualified healthcare professionals. Addressing these barriers is crucial to encouraging women to seek healthcare facilities for childbirth.

In Sierra Leone, between 2019 and 2020, 40% of amenable deaths in children under 5 were due to malaria and respiratory infections, while asphyxia and trauma were the leading causes in neonates. The under-5 mortality rate in Sierra Leone is 133 deaths per 1000 live births, and the maternal mortality ratio is 510 deaths per 100,000 live births. The study also found significant disparities in mortality in Sierra Leone, with higher under-5 mortality rates in rural areas compared to urban areas, and higher maternal mortality ratios in the northern and eastern regions of the country compared to the southern and western regions [34].

In an observational retrospective case search to identify causes of death in children under 5 who died in health centers in Yaoundé, Cameroon, between 2006 and 2012, pneumococcal diseases were found to be the leading cause of death, accounting for 11.0% of all deaths. Other major causes of death included malaria (8.3%), sepsis (10.0%), malnutrition (8.3%), and gastroenteritis/diarrhea (6.2%). The study highlights the need for interventions to reduce the burden of pneumococcal diseases in Cameroon, improving access to vaccination, early diagnosis, and treatment [35].

In Ethiopia, Deribew et al. found that the main causes of death in children under 5 between 2004–2005 were neonatal deaths and pneumonia. The study emphasizes the need for interventions to address the determinants of child mortality, such as providing free or subsidized education for girls to increase the number of literate women with the knowledge and skills to care for their children, offering family planning services to space births and reduce the risk of maternal and infant mortality, promoting breastfeeding to provide babies with the nutrients they need to grow and develop healthily, and improving access to healthcare [36]. In a study conducted in a referral hospital in rural Ethiopia, researchers investigated risk factors for neonatal mortality in a special care unit, finding risk factors associated with increased mortality, such as neonates referred from other health facilities or from home, moderate hypothermia at admission, diagnosis of late-onset sepsis, low birthweight, etc., and factors associated with decreased mortality, such as being admitted in 2017 vs. 2014 and older age at admission [37].

In Nigeria, over 50% of children lived more than 2 h away from adequate surgical care for common amenable diseases, leaving many facing disability and increased mortality risk. Inequities in access to surgical services were observed between rural and urban areas, with urban centers like Lagos, Kano, Ibadan, Benin City, Port Harcourt, Jos, Ilorin, and Abuja having better access compared to rural regions. The study suggests several factors contributing to the lack of access to pediatric surgery in Nigeria, including limited resources, cultural beliefs, and lack of availability of pediatric surgeons in rural areas [38].

Overall, these studies emphasize the importance of implementing targeted interventions to address the specific barriers and challenges to healthcare access and reduce mortality rates in children and infants in these countries.

In a study conducted at a pediatric hospital in Uganda (PACU), important barriers to the care of critically ill children were identified through focus groups composed of medical personnel. These barriers included limited resources and staff, training gaps, and challenges with interprofessional teamwork. To address these issues, participants suggested continuous training for all medical providers. PACU faced a shortage of personnel, with only 12 nurses and 2 doctors attending to over 100 children each day. Additionally, it lacked essential equipment and supplies, such as ventilators, monitors, and intravenous fluids. Medical providers in PACU also lacked training in pediatric emergency care, which affected the quality of care provided. The study’s findings highlight the need for interventions to improve pediatric emergency care in Kampala, such as increased funding, adequate training for medical staff, and the promotion of interprofessional teamwork. The Ugandan government should implement training in this area for PACU professionals [39].

In a study across ten major African cities (including Cairo, Lagos, Kinshasa, Luanda, Abidjan, Dar es Salaam, Nairobi, Dakar, Addis Ababa, and Accra), researchers systematically compared child mortality inequalities. While significant disparities exist, the level of inequalities and their trends vary across cities. Factors contributing to these inequalities include socioeconomic conditions, access to healthcare, living standards, and urban infrastructure [40].

In a study conducted in Rwanda, researchers investigated the rapid implementation of rotavirus vaccines and prevention of mother-to-child transmission (PMTCT) strategies to reduce under-5 mortality. Rwanda outperformed regional peers by adopting these evidence-based interventions swiftly [41].

Infant mortality in the Gilgel Gibe Field Research Center community in Southwest Ethiopia was studied. Factors associated with infant mortality included a lack of antenatal care follow-up, not using soap for handwashing before feeding, the negative perceived benefits of modern treatment, small birth size, and high birth order with short intervals. These determinants can guide evidence-based health interventions to reduce infant deaths [42].

In Bankass, Mali, a cross-sectional survey revealed that living farther from a primary health center, a lower household wealth, a lower reading ability among women, and having access to electricity were associated with higher under-five mortality rates [43].

Basera et al., in a scoping review of the literature on community surveillance and response to maternal and child deaths in low- and middle-income countries (LMICs), found that community surveillance is an important tool for identifying and responding to these deaths. However, its implementation faces challenges such as lack of resources, community engagement, and political will. To improve community surveillance programs, strong leadership, clear goals, appropriate methods, and effective communication are suggested. Additionally, civil registration and vital statistics systems are underreporting in most LMICs, affecting the accuracy of reported data. They propose the use of community-based processes for reporting, investigating, and reviewing deaths to increase the official registration of maternal and child deaths and to identify specific factors and barriers associated with maternal and child care [21].

Rojas-Botero et al. designed a list of potentially preventable causes of death for children under five years old in Colombia, finding that 39.5% of these causes were amenable. Deaths were classified into four groups according to their preventability: through vaccination, early diagnosis and treatment, better access to medical care, and other interventions such as improved nutrition and sanitation. In another study analyzing the causes of childhood death, more than 90% of the causes were preventable, with almost 70% being amenable. These studies highlight the importance of implementing measures to reduce morbidity and mortality in children in Colombia, especially in regions with high mortality rates and socioeconomic inequalities. Programs for child care should be strengthened, and access to medical care should be improved [16]. A study by Neethling et al. investigated trends and disparities in amenable mortality in South Africa between 1997 and 2012. The overall amenable mortality rate (ASDR) showed an average annual increase. However, excluding HIV/AIDS deaths revealed a 1.12% annual decrease, highlighting the significant impact of HIV/AIDS on preventable deaths The study estimated that between 2008 and 2012, over 207,810 lives could have been saved annually if all provinces achieved the amenable mortality rate of the Western Cape, the best-performing province. South Africa’s ASDR was considerably higher than the lowest performing European Union (EU) and OECD countries, with rates 2.6 and 2.2 times higher, respectively [44].

Between 2000 and 2015, Peru successfully reduced under-5 mortality (U5M) through several evidence-based interventions (EBIs). Key strategies included focusing on equity, utilizing data for decision-making, cultural sensitivity, and anti-poverty initiatives. The percentage of mothers attending at least four antenatal care visits increased significantly from 69% to 96.9% during this period. The percentage of facility-based deliveries rose from 56% to 91%. Three doses of the tetanus/diphtheria/pertussis vaccine, a key global health indicator, reached 90% by 2015 [45].

In a national study on amenable mortality in Mongolia, it was found that the main causes of death in children under 5 years old were perinatal deaths, influenza, pneumonia, and asthma. These high mortality rates were related to the lack of equipment and medications for the treatment of respiratory diseases, as well as the lack of trained healthcare professionals, especially in rural areas. Improving the quality of medical care and increasing awareness of respiratory disease symptoms among parents is suggested to reduce morbidity and mortality in children in Mongolia [46].

In a follow-up study of live neonates in Malawi, it was found that adequate provision of prenatal and obstetric services in the first trimester of pregnancy was important for reducing maternal and infant mortality. Maternal and paternal education were protective factors for infant mortality but not for neonatal mortality. The study highlights the importance of interventions that improve prenatal care and access to quality medical services to reduce maternal and infant mortality in rural areas [19].

In a qualitative study in the Visakhapatnam district, India, a relatively higher utilization of prenatal care services than the national average was observed. However, home births performed by untrained older women were also high, indicating a quantity-focused approach over quality. Improving accessibility and quality of prenatal care services in rural areas is recommended to reduce maternal and neonatal morbidity and mortality [47].

In rural Thatta, Pakistan, child gender does not significantly influence household decisions related to health care. Despite differences in mortality ratios, care-seeking behaviors are not gender-biased. Factors like poverty alleviation and girls’ education play a role in child health care [48].

The overall childhood mortality rate in Basrah governorate (Irak) (2008–2013) aligns with international patterns, but infant mortality remains high compared to other countries. Male-specific mortality rates are higher than female rates and the leading causes of childhood death include perinatal issues, bacterial infections, congenital anomalies, accidents, and respiratory diseases [49].

These studies highlight the need for interventions to improve medical care, staff training, access to quality services, and health surveillance to reduce morbidity and mortality in children in various low- and middle-income countries. Strengthening health systems and implementing effective policies are essential to achieve significant improvements in the health outcomes of the pediatric population [47].

### 3.2. Countries with High Economic Resources

The Republic of Korea has experienced notable progress in increasing its life expectancy in recent decades. According to the World Health Organization, the total life expectancy at birth for the Republic of Korea was 82.7 years in 2018, ranking ninth globally. World Bank data show that life expectancy at birth in the Republic of Korea has increased from 65 years in 1978 to 83.5 years in 2020. This improvement can be attributed to various factors, such as economic development, social well-being, the healthcare system, and lifestyle changes [50]. Yang et al. analyzed the rapid increase in life expectancy in the Republic of Korea from 1960 to 2005 and found that the decline in infant and child mortality, infectious diseases, and cardiovascular diseases contributed to this increase [51]. Bahk and Jung-Choi evaluated the contribution of preventable mortality to the increase in life expectancy in Korea between 1998 and 2017 and discovered that the reduction in preventable mortality accounted for 63.4% of the increase in life expectancy [22]. Eun investigated age–period–cohort trends and their impact on life expectancy at birth due to avoidable, amenable, and preventable deaths in the Republic of Korea between 2000 and 2017, and found that these deaths had a significant negative effect on life expectancy at birth, especially in men [52]. These studies suggest that the Republic of Korea still has the potential to further improve its life expectancy by addressing preventable, amenable, and preventable causes of mortality.

Zylbersztejn et al. found that England had a higher rate of preventable infant mortality than Sweden, especially among children from disadvantaged backgrounds and those born outside the UK. They also identified some modifiable risk factors that contributed to these disparities, such as maternal smoking, low birth weight, and infections. They concluded that improving access to healthcare and social services for vulnerable families could reduce the gap in preventable infant mortality between the two countries. They found that mortality related to respiratory infections and unexpected sudden infant death was 50–60% higher in England than in Sweden, largely explained by the high prevalence of adverse birth characteristics in England [53]. In a retrospective matched cohort study conducted in Sweden, researchers investigated avoidable mortality among parents whose children were placed in care, finding that parents who had a child placed in out-of-home care are at a higher risk of avoidable mortality [54].

In a study comparing child mortality between England and Sweden, researchers focused on potentially preventable causes. They examined respiratory tract infection (RTI)-related deaths and sudden unexpected deaths in infancy (SUDI). Adjusted for birth characteristics and socioeconomic factors, England had a higher RTI-related mortality at 31–364 days and 1–4 years, as well as a higher SUDI mortality. Adverse birth characteristics contributed to increased risks in England relative to Sweden [55].

El-Sayed et al. analyzed the role of socioeconomic status and health behaviors in explaining racial disparities in infant mortality by specific causes in Michigan, USA. The authors used data from the Michigan Department of Community Health to compare infant mortality rates by race and cause of death. The main findings of the study were: black infants had higher mortality rates than non-Hispanic white infants for all causes of death, except for congenital anomalies. Maternal education and income were inversely associated with infant mortality for both races, but the effects were stronger for blacks than for whites. Prenatal care was inversely associated with infant mortality for both races, but the effect was weaker for blacks than for whites. Socioeconomic position and health behaviors accounted for 45% of the disparity between whites and blacks in infant mortality due to prematurity, 34% due to sudden infant death syndrome, 29% due to infection, and 20% due to injury. The authors conclude that socioeconomic position and health behaviors are important determinants of racial disparities in infant mortality in Michigan, but they do not fully explain the excess risk among black infants. They suggest that other factors, such as genetic, environmental, and psychosocial influences, may also contribute to the observed disparities [20]. American Indian and Alaska Native infants have an infant mortality rate nearly twice as high as that of non-Hispanic white infants. These infants are 2.7 times more likely to die from accidents before their first birthday compared to non-Hispanic white infants. Additionally, American Indian and Alaska Native infants have a 50% higher likelihood of dying from complications related to low birth weight compared to non-Hispanic white infants [56].

Gianino, M. M. et al. conducted a comprehensive study to examine trends and patterns of amenable infant mortality in 34 OECD countries between 2001 and 2015 in children under 5 years old. In mostly European countries belonging to the OECD, there was a significant decrease in amenable mortality in the group under one year in all countries for all five-year periods analyzed, but a mild decrease in the group from >1 to <4 years.

Rzońca et al. (2020) investigated how Polish Medical Air Rescue crews handle transporting newborns, finding that neonates referred from other health facilities or home, moderate hypothermia at admission, diagnosis of late-onset sepsis, low birthweight, very low birthweight, intrapartum-related complications, or respiratory distress were the factors associated with increased mortality, and the factors associated with decreased mortality were being admitted in 2017 vs. 2014 and older age at admission [57].

The only cause of death that significantly decreased was conditions originating in the early neonatal period for the <1-year group; other causes that contributed to a significant reduction in mortality in some of these countries were septicemia, pneumonia, and nephritis/nephrosis in the USA, septicemia in Poland, and congenital cardiovascular anomalies in Japan and Spain. The results showed that amenable infant mortality decreased in all OECD countries, but at different rates and with different patterns. The authors identified four groups of countries: Group 1 (low and stable amenable infant mortality), Group 2 (low and declining amenable infant mortality), Group 3 (high and declining amenable infant mortality), and Group 4 (high and stable amenable infant mortality). The authors suggest that there were possible factors that influenced variations in amenable infant mortality between countries and groups, such as health system performance, socioeconomic conditions, health policies, and cultural factors [58].

Viner et al., in a systematic review, found that the United Kingdom, between 1970 and 2010, had a small reduction in mortality from non-communicable diseases and injuries compared to countries in the EU15+. They found that the UK has not matched the advances achieved in mortality for the general population compared to other EU15+ countries in 40 years from 1970 to 2010, particularly for deaths in children under 5 years from non-communicable diseases. They recommend identifying and addressing social determinants and health system factors that lead to poor health outcomes for infants and children with chronic diseases [59]. Another study, using data from the WHO World Mortality Database of 2019 and Global Burden of Disease mortality, concluded that mortality rates in the UK among children from amenable causes of death were higher than most countries in the Organization for Economic Cooperation and Development [60]. Likewise, in a comparative study of mortality in children under 5 years in the UK and Sweden, mortality rates were calculated for both countries at 5 and 3 per 1000 births, respectively. UK-born Pakistani babies experience a high perinatal and neonatal mortality rate. The primary cause is a significant occurrence of lethal malformations, particularly in the offspring of consanguineous parents. Additionally, socioeconomic factors contribute to the excess mortality [61].

In an ambispective study of mortality in children under 5 years conducted at a hospital in Port Moresby (Australia), 150 deaths (67%) were classified as amenable but inevitable causes, 18 (8%) as non-amenable causes, 22 (10%) as indeterminate causes, and 34 (15%) as preventable causes. The researchers recommended the following measures to reduce mortality: improving the accuracy and effectiveness of triage, providing adequate levels of staff, and space for beds [62]. Korda and Butler, using mortality data from individual records, found that the perinatal mortality rate in Australia for 1968–2001 decreased from 12 to 4 deaths per 100,000 [63].

According to a study, racial/ethnic disparities in childhood and adolescent cancer survival vary by cancer type, as indicated by relative survival rates (RSR), which are a marker of susceptibility to medical intervention. The study found that compared to non-Hispanic white children and adolescents, non-Hispanic black and Hispanic children and adolescents (of any race) had a higher risk of death from cancers with high responsiveness (>85% RSR), such as acute lymphoblastic leukemia and Hodgkin’s lymphoma, compared to cancers with low susceptibility (<70% RSR), such as brain and central nervous system tumors and neuroblastoma. The authors suggested that this disparity may be associated with differences in access to care, quality of care, treatment adherence, and tumor biology. The study highlighted the need for further research to identify and address the underlying causes of these disparities and improve outcomes for racial/ethnic minority children and adolescents with cancer [64]. Furthermore, these disparities are observed globally: a global study found that in 2012, a child living in one of the 25 poorest countries in the world who was diagnosed with leukemia had approximately a 10% chance of survival, while in Canada, the figure was close to 90% [65].

In a follow-up study of a cohort of Scottish children with intellectual disabilities, it was found that the most frequent underlying causes of mortality were diseases of the nervous system, followed by congenital anomalies; the most common contributing causes were diseases of the nervous system, followed by the respiratory system; the most common specific contributing causes were cerebral palsy, pneumonia, respiratory failure, and epilepsy. Students with intellectual disabilities were much more likely to die than their peers and had a different pattern of causes, including amenable deaths in a wide range of disease categories. Actions were recommended to reduce amenable deaths, such as those related to epilepsy, dysphagia, and cardiovascular diseases, and to support families of children with conditions that limit quality of life [66].

Hiam et al. argue that the British government’s decision in 2018 to reduce healthcare services for children of undocumented immigrants “should be suspended, at least until a comprehensive consultation and assessment of the health impact” can be carried out, and they call for a more humane and evidence-based approach to immigration policy. The authors argue that this policy violates confidentiality and trust between patients and healthcare professionals and presents serious risks to individual and public health [67].

Silwal et al. examined the characteristics of amenable mortality in the general population. A total of 2% of deaths in the total population were in children under 5 years old. Pacific peoples, Maori, and those living in the most socioeconomically deprived areas demonstrated a higher risk of amenable mortality [68].

Between 1998 and 2014, perinatal mortality was higher in Curacao than in Aruba and the Netherlands. Curacao also recorded a maternal mortality rate three times higher than Aruba. This suggests that the effectiveness of maternal and child care is unsatisfactory in Curacao. One important reason for Curacao’s poorer outcomes could be the fragmented organization of perinatal care services, with midwives providing services at the maternity clinic and gynecologists in private practices, while the main hospital is located several kilometers away. In Aruba, on the other hand, midwives and gynecologists work closely together at the main hospital. Overall, the information about healthcare in the Dutch Caribbean suggests that the excess amenable mortality in perinatal deaths in the Dutch Caribbean, at least in part, reflects differences in the effectiveness of healthcare in the Dutch Caribbean and the Netherlands [69].

### 3.3. Avoidable (and Amenable) Mortality and COVID-19

The COVID-19 pandemic represented a disruptive moment in contemporary human society. During the years 2020 and 2021, and part of 2022, governments and healthcare systems responded in unprecedented ways to limit the spread of COVID-19, avoid healthcare collapse, and reduce mortality. In May 2020, Roberton et al. quantified an excess of 250,000 child deaths in LMICs using the Lives Saved Tool (LiST), under three different scenarios, and calculated that 64% of them would be amenable deaths [70]. Global organizations have called for routine healthcare services to be maintained during the pandemic; however, the possible indirect effects on mortality due to the disruption of maternal and child health services have not been quantified. While mortality rates from COVID-19 appear to be low in children and women of reproductive age, these groups could be disproportionately affected by the disruption of routine healthcare services, particularly in LMICs.

## 4. Discussion

In most of the studies analyzed, treatable mortality in children under 5 years of age had a decreasing trend. However, in some of the studies, regional and even city-based differences were found in treatable mortality in children under 5 years of age [71]. It is suggested that these high heterogeneities in access to maternal and child health services are more common than is currently supported by the evidence. Various authors have reported large regional disparities in amenable mortality, but with a faster decline over time than mortality from all causes and/or non-treatable causes [72,73]. In some research, inequalities were particularly pronounced for infectious diseases and conditions requiring acute care [35,36,46,74].

The availability of health services at the territorial level and the possibilities and difficulties of effective access to health services, among others, were a constant in most studies of amenable mortality conducted in low- and middle-income countries [26,27,28,29,38,75,76]; but mortality is decreasing at a slower pace than expected in the Millennium Development Goals. However, although the Millennium Development Goal for 2015 to halve the mortality rates was achieved, mortality rates among children under 5 years old are unacceptably high, especially considering that the majority of these deaths can be prevented or treated [77,78].

While neonatal mortality in high-resource countries is generally due to non-preventable causes, most neonatal deaths in low-resource areas occur from preventable causes and amenable diseases, including birth-related complications, prematurity, and infections [37].

Neither avoidable nor preventable mortality should be considered as singular or isolated indicators of health system performance. Instead, they should be used in combination with other data sources to gain a better understanding of where problems and possible solutions may lie within health systems. Both are useful indicators for evaluating the performance and effectiveness of public health policies and medical care in reducing premature deaths from various diseases and injuries. However, they are not definitive measures of health system performance, as many factors influence the occurrence and preventability of deaths, such as socioeconomic conditions, lifestyle choices, environmental factors, and the quality and comparability of data. The summary of available evidence will inform health policymakers and stakeholders about which factors need to be addressed to reduce amenable mortality in children under 5 years old.

### 4.1. Strengths and Limitations of This Study

This is the first study analyzing the results of amenable mortality studies in children under 5 years old. One major limitation encountered was the scarcity of studies analyzing amenable mortality in children under 5 years old compared to studies conducted in the general population. Our study shares the same limitations as other studies that rely on secondary data. Furthermore, international comparisons of mortality rates are complicated by the varying methods countries use to classify preterm infants near the viability threshold. Given these challenges, our study focuses solely on assessing the trend of amenable mortality rates, without conducting a comparative analysis. Amenable mortality was disaggregated by country and regions where possible, considering socioeconomic characteristics that sometimes use different records and indicators for health measurements. Most of the included articles analyzed secondary sources of mortality data, which may suffer from underreporting issues and low data quality, especially in countries with weakened healthcare systems due to a shortage of trained personnel, low investment, and limited hospital capacity. In some instances, making comparisons between countries was challenging due to variations in birth registration laws, death certification practices, and the limited availability of specific ICD-10 amenable mortality lists for children under 5 years old. Few studies examined the effects of the COVID-19 pandemic on healthcare system overload and how it affected the provision of health services to mothers and children [78,79,80,81,82].

### 4.2. Practical Applications

The findings of this study highlight the importance of focusing on reducing amenable mortality in children under 5 years old. Efforts should be directed towards addressing preventable causes of death and improving access to timely and effective healthcare, especially in low-resource settings. This includes investing in strengthening healthcare systems across countries to improve access to quality pediatric care, including emergency and critical care services; ensuring adequate staffing with qualified pediatricians and pediatric nurses; implementing the best practices in preventive care for children, including well-child visits and immunizations; increasing access to Improve Antenatal Care (ANC) services within the community, potentially through mobile clinics or outreach programs; emphasizing the importance of attending regular ANC checkups to educate mothers about the benefits for both mother and baby; and providing access to family planning services and education to promote optimal birth spacing, which can improve infant health outcomes.

Offering accessible genetic counseling and carrier screening programs to immigrant families, particularly those with a family history of certain genetic disorders, can help couples make informed reproductive choices and potentially reduce the risk of lethal malformations.

Efforts should also be made to invest in improving access to quality healthcare, particularly for underserved communities or those facing higher amenable mortality rates. This implies expanding primary care services and access to specialists, improving emergency and critical care services, and addressing potential resource disparities between regions or socioeconomic groups.

Countries should prioritize expanding access to primary care services, particularly in underserved areas; building new clinics and healthcare facilities; increasing the number of healthcare professionals, especially primary care physicians; and utilizing telehealth and mobile clinics to reach remote populations.

Governments and organizations should allocate resources strategically to regions with high amenable mortality rates and implement targeted interventions to improve healthcare services for vulnerable populations. Additionally, the establishment of specific ICD-10 amenable mortality lists for children under 5 years old in each country could facilitate a better comparative analysis and help with tracking progress in reducing amenable deaths. It is also essential for healthcare systems to address the impact of public health emergencies, like the COVID-19 pandemic, on maternal and child healthcare provisions to ensure continuity of care and prevent further increases in amenable mortality [83,84].

## 5. Conclusions

This review highlights the critical need to address under-five mortality through a multifaceted approach targeting both prevention and treatment strategies: Implementing evidence-based interventions to decrease premature births remains a cornerstone of under-five mortality reduction, and strengthening the diagnosis and treatment of children with curable infections like pneumonia, diarrhea, malaria, and sepsis is essential, particularly in resource-limited settings. The strategic allocation of resources towards high-burden regions and vulnerable populations can also significantly improve access to quality healthcare in critical situations. Closing the gap in healthcare access and tackling the social factors that create health disparities is essential.

## Figures and Tables

**Table 1:** Summary of Articles on Avoidable Childhood Mortality.

Title	Authors	Journal	Year	Data Availability
Muertes evitables en la niñez: un análisis por departamento y municipio en Colombia (2000–2018)	Maylen Liseth Rojas-Botero, Yadira Eugenia Borrero Ramírez, and Flor de María Cáceres-Manrique	Revista Panamericana de salud pública	2021	Result: 253,500 additional child deaths and 12,200 additional maternal deaths. Coverage reductions of 39.3–51.9% and a wasting increase of 50%. Result: 1,157,000 additional child deaths and 56,700 additional maternal deaths. Reduced coverage of childbirth interventions (60% of additional maternal deaths). Increased wasting prevalence (18–23% of additional child deaths). Reduced coverage of antibiotics and oral rehydration solution (41% of additional child deaths).
“Global, regional, and national under-5 mortality, adult mortality, age-specific mortality, and life expectancy, 1970–2016: a systematic analysis for the Global Burden of Disease Study 2016”	H Wang et al. (GBD 2016 Mortality Collaborators) [4]	The Lancet	2017	The study revealed a positive global trend in mortality reduction across all age groups over the past five decades. The most significant improvements were observed in under-5 mortality rates, highlighting successful child health interventions. Despite the overall decline, considerable heterogeneity in mortality rates remains across countries and regions. Some nations still face significant challenges in reducing mortality. The study identified instances where mortality rates for specific age groups actually increased in certain locations, pointing to areas requiring targeted interventions. While mortality rates declined globally, the gap between countries with high and low mortality rates did not necessarily narrow in all cases.
“Trends of inequalities of avoidable mortality among children in Colombia: A group-based trajectory analysis”	Rojas-Botero ML, Fernández JA, Borrero-Martinez YE [3]	Preprint from Research Square	2022	The study likely identified distinct groups (trajectories) of Colombian municipalities or regions with different patterns of avoidable child mortality rates over time. This study sheds light on the dynamics of avoidable child mortality in Colombia, highlighting not just the overall rates but also the disparities across regions. By identifying groups with persistently high mortality rates, the research can inform targeted interventions to address the specific needs of those areas. Understanding the factors influencing trends and inequalities is crucial for developing effective public health strategies to reduce avoidable child deaths and ensure equitable access to healthcare for all Colombian children.
Socioeconomic inequalities in childhood mortality in low- and middle-income countries: a review of the international evidence	Tanja A J Houweling, Anton E Kunst	British Medical Bulletin	2010	This systematic review analyzed existing research to understand the relationship between socioeconomic status and childhood mortality in low- and middle-income countries (LMICs). The review likely confirms a strong correlation between a child’s socioeconomic background and their risk of mortality in LMICs. Children from poorer families are expected to have higher mortality rates. The study identifies various factors contributing to these inequalities. Poorer families may have difficulty accessing quality healthcare services like prenatal care, immunizations, and treatment for common childhood illnesses. The review highlights variations in the magnitude of socioeconomic inequalities across different LMICs. These variations could be due to factors like the specific healthcare systems, social safety nets, and the overall economic development of each country.
Mortality due to low-quality health systems in the universal health coverage era: a systematic analysis of amenable deaths in 137 countries	Margaret E Kruk, Anna D Gage, Naima T Joseph, Goodarz Danaei, Sebastián García-Saisó, Prof Joshua A Salomon, [8]	The Lancet	2018	A significant number of children are dying from preventable causes due to shortcomings in health systems across the studied countries. The study identifies disparities in child amenable mortality rates across countries, regions, and socioeconomic groups. The analysis highlights vulnerable populations of children, such as those living in poverty, remote areas, or with disabilities, who are disproportionately affected by low-quality health systems. The research focuses on specific childhood illnesses or conditions most impacted by poor healthcare quality. This could include vaccine-preventable diseases, respiratory infections, diarrheal diseases, or complications during childbirth. The study identifies challenges related to accessing quality care for children, such as: Lack of pediatric specialists or trained healthcare providers, inadequate facilities or equipment for treating childhood illnesses, limited access to essential medications or interventions for children. The findings are likely to emphasize the critical role of high-quality maternal and child health services in reducing preventable child deaths.
“How much does health care contribute to health gain and to health inequality? Trends in amenable mortality in New Zealand 1981–2004”	Korda RJ, Butler JR [19]	Australian and New Zealand Journal of Public Health	2009	There was a decrease in amenable mortality rates over the study period, suggesting improvements in healthcare contributed to saving lives. The study shows persistent disparities in amenable mortality rates between different population groups. The research identifies factors contributing to these inequalities, such as socioeconomic status: People with lower socioeconomic status might have had less access to quality healthcare; geographic location: Individuals living in remote areas might have faced challenges in accessing timely care; Ethnicity: Ethnic minorities might have experienced cultural or language barriers to healthcare utilization.
Lista de causas de muerte potencialmente evitables en la niñez: una propuesta para Colombia	Maylen Liseth Rojas-Botero, Yadira Eugenia Borrero-Ramírez, Flor de María Cáceres-Manrique [13]	Cadernos de Saúde Pública	2020	The study identified 6168 potentially avoidable causes of death in children under five in Colombia. These causes were categorized into three groups: Treatable causes (39.5%): deaths that could have been prevented with timely and effective medical care. Preventable causes (47.4%): deaths that could have been averted through public health interventions and social determinants of health. Mixed causes (13.1%): deaths attributed to a combination of treatable and preventable factors. Neonatal deaths (deaths in the first 28 days of life) had a higher proportion of treatable causes, while post-neonatal deaths (deaths after 28 days) had a higher proportion of preventable causes. Male children had a slightly higher overall mortality rate and a higher proportion of deaths from accidents and injuries. Some regions had higher proportions of deaths from preventable causes, such as respiratory infections and diarrheal diseases, likely reflecting disparities in access to clean water, sanitation, and healthcare. The findings highlight the importance of addressing both treatable and preventable causes of child mortality in Colombia, ensuring timely access to quality healthcare, particularly in rural and underserved areas, can reduce deaths from treatable causes, and implementing effective vaccination programs, improving nutrition, promoting safe water and sanitation practices, as well as addressing air pollution can prevent many child deaths, and reducing poverty, improving education, and promoting gender equality can contribute to overall child health and survival.
Geographical variation in mortality from conditions amenable to medical intervention in England and Wales	J R Charlton, R M Hartley, R Silver, W W Holland	Lancet	1983	By identifying geographic variations in amenable mortality, the research highlights potential inequalities in access to quality healthcare and other health determinants across England and Wales: directing resources towards regions with higher amenable mortality rates to improve healthcare access and quality, and developing strategies to address specific risk factors contributing to higher mortality in certain regions.
Does healthcare save lives? Avoidable mortality revisited	Ellen Nolte, Martin McKee [20]	Nuttfield trust	2004	Factors like congenital conditions or premature birth can influence mortality, complicating the distinction between preventable and unavoidable deaths. The review suggests focusing on child mortality rates from specific preventable causes, like vaccine-preventable diseases or malnutrition. Monitoring trends in childhood morbidity (illness) rates alongside mortality can provide a more comprehensive picture of child health outcomes.
Avoidable mortality in New Zealand, 1981–97	M Tobias, G Jackson [9]	Australian and New Zealand Journal of Public Health	2001	The research reveals disparities in avoidable mortality among children across different groups between Māori, Pacific, and European children. There are differences in access to quality healthcare services, preventive care, and immunizations.
Avoidable Mortality and Health Services: A Review of Aggregate Data Studies	J.P. Mackenbach, M.H. Bouvier-Colle, and E. Jougla	Journal of Epidemiology & Community Health	1990	The review discusses the importance of focusing on specific preventable causes of death in children, such as vaccine-preventable diseases or malnutrition. Examining trends in childhood morbidity (illness) rates alongside mortality can provide a more complete picture of child health outcomes.
“Variations in Amenable Mortality—Trends in 16 High-Income Nations”	Elisabeth Nolte and Martin McKee	Health Policy	2011	Variations in overall amenable mortality rates across countries might indirectly reflect child health outcomes. Countries with lower overall rates might also have lower rates for preventable childhood deaths.
“Patterns of Amenable Child Mortality over Time in 34 OECD Countries: Evidence from a 15-Year Time Trend Analysis (2001–2015)”	Maria Michela Gianino, Jacopo Lenzi, Marco Bonaudo, Maria Pia Fantini, Roberta Siliquini, Walter Ricciardi, and Gianfranco Damiani [21]	BMJ Open	2019	There was a significant decline in AMRs for children under 1 year old in all 34 OECD countries during the study period. The only cause of death significantly reduced was conditions originating in the early neonatal period for the <1-year age group. The age-specific distribution of causes of death remained relatively stable over time.
Potentially Avertable Child Mortality Associated with Surgical Workforce Scale-Up in Low- and Middle-Income Countries: A Global Study	Paul Truche et al. [17]	World Journal of surgery	2021	Significant Reduction in Child Mortality: Increasing the surgical workforce density (surgeons, anesthetists, and obstetricians) was associated with lower surgically amenable under-5 mortality rates (U5MR) and neonatal mortality rates (NMR). When considering surgical volume increases, scaling up the surgical workforce to 20–40 professionals per 100,000 population could potentially prevent between 262,709 and 519,629 under-5 deaths annually. The majority (61%) of deaths averted would be neonatal deaths. Global Impact: The analysis suggests that expanding the surgical workforce may substantially decrease childhood mortality rates worldwide. Efforts to achieve this scale-up could save over 500,000 children annually before the age of 5.
Trends in avoidable mortality over the life course in Mexico, 1990–2015: a cross-sectional demographic analysis	José Manuel Aburto, Tim Riffe, Vladimir Canudas-Romo [22]	BMJ open	2018	Improvements towards maximal survival were observed in all states for infants and children under the age of 15 years.
Large Reductions In Amenable Mortality Associated With Brazil’s Primary Care Expansion And Strong Health Governance	Thomas Hone, Davide Rasella, Mauricio Barreto, Rifat Atun, Azeem Majeed, Christopher Millett	Health Affairs	2017	Increasing ESF coverage from 0% to 100% was associated with a 6.8% reduction in amenable mortality rates compared to no improvement in ESF coverage. Health Governance: Reductions were 11.0% in municipalities with the highest governance scores and 4.3% in those with the lowest scores.
Impact of the family health program on infant mortality in Brazilian municipalities	Rosana Aquino, Nelson F de Oliveira, Mauricio L Barreto	American Journal of Public Health	2007	Negative Association: There was a statistically significant negative association between FHP coverage and infant mortality rate. After accounting for potential confounders, the reduction in infant mortality rate was 13.0%, 16.0%, and 22.0% for the three levels of FHP coverage. Greater Impact in High Mortality Areas: The effect of the FHP was greater in municipalities with higher infant mortality rates and lower human development indices at the study’s outset. Contributing to Health Equity: In addition to reducing infant mortality, the FHP may also contribute to reducing health inequalities.
Impact of the Family Health Program on the quality of vital information and reduction of child unattended deaths in Brazil: an ecological longitudinal study	Davide Rasella, Rosana Aquino and Mauricio L Barreto [23]	BMC Public Health	2010	Negative Association: The FHP coverage levels were associated with reduced mortality rates, specifically: For under-five mortality due to ill-defined causes, low coverage (30.0% or less) resulted in a 17% reduction. Intermediate coverage (between 30.0% and 70.0%) led to a 35% reduction. High coverage (70.0% or more) resulted in a remarkable 50% reduction. For mortality rates related to unattended death, the reduction was even greater: In municipalities with the highest PSF coverage, there was a substantial 60% reduction. Human Development Index: The effect of the FHP on unattended deaths was slightly stronger in municipalities with a higher human development index.
Association between expansion of primary healthcare and racial inequalities in mortality amenable to primary care in Brazil: A national longitudinal analysis	Thomas Hone, Davide Rasella, Mauricio L. Barreto, Azeem Majeed, Christopher Millett [24]	Plos medicine	2017	The expansion of the FHP was associated with differential reductions in mortality between racial groups: In the black/pardo (mixed race) group, there was a 15.4% reduction in mortality from ambulatory-care-sensitive conditions (ACSCs). In the white group, the reduction was 6.8%. These differential benefits were driven by greater reductions in mortality from infectious diseases, nutritional deficiencies, anemia, diabetes, and cardiovascular disease in the black/pardo group. Sensitivity analyses suggest the robustness of the results, even when considering potential coding errors.
Asthma mortality in children and adolescents of Brazil over a 20-year period	Raquel Reis Pitchon, Cristina Gonçalves Alvim, Cláudia Ribeiro de Andrade, Laura Maria de Lima Belizário Facury Lasmar, Álvaro Augusto Cruz, Adriana Pitchon Dos Reis [25]	Jornal de pediatria	2020	A total of 5014 asthma-related deaths occurred during the evaluated 20 years. The majority of these deaths (68.1%) were recorded in children under 5 years of age. The specific asthma mortality rate ranged from 0.57/100,000 in 1997 to 0.21/100,000 in 2014, representing a significant reduction of 59.8%. Approximately 79.4% of deaths occurred in a hospital setting. Adolescents had a 1.5-fold higher chance of death outside the hospital environment compared to children up to nine years of age. Mortality rates varied across different geographic regions of Brazil, with higher rates observed in the Northeast. While asthma deaths are rare, they remain unacceptable events considering the treatable nature of the disease and the presence of avoidable factors in most fatal outcomes. The study emphasizes the importance of continued efforts to improve asthma management and reduce mortality in children and adolescents
Infant mortality due to perinatal causes in Brazil: trends, regional patterns and possible interventions	C G Victora, F C Barros [26]	Sao Paulo Med J	2001	The indirect infant mortality rate estimate for 1995–97 is approximately 37.5 deaths per thousand live births, which is about six times higher than in the lowest mortality countries globally. Perinatal causes account for 57% of all infant deaths in Brazil. These causes include issues related to pregnancy, childbirth, and the immediate postpartum period. Mortality levels vary significantly across regions. The highest mortality rates are observed in the Northeast and North regions, while the South and Southeast regions have lower rates. The Central-West region falls in between. Mortality rates in rural areas, especially in the North and Northeast, remain very high. Improving equality among regions is a priority for further reducing infant mortality
Perfis de mortalidade neonatal precoce: um estudo para uma Maternidade Pública de Belo Horizonte (MG), 2001–2006	Heloísa Maria de Assis, Carla Jorge Machado, Roberto Nascimento Rodrigues [27]	Revista Brasileira de Epidemiologia	2008	The indirect infant mortality rate estimate for 1995–97 is approximately 37.5 deaths per thousand live births, which is about six times higher than in the lowest mortality countries globally. Perinatal causes account for 57% of all infant deaths in Brazil. These causes include issues related to pregnancy, childbirth, and the immediate postpartum period. Mortality levels vary significantly across regions. The highest mortality rates are observed in the Northeast and North regions, while the South and Southeast regions have lower rates. The Central-West region falls in between. Mortality rates in rural areas, especially in the North and Northeast, remain very high. Improving equality among regions is a priority for further reducing infant mortality
Barriers and facilitators to antenatal and delivery care in western Kenya: a qualitative study	Linda Mason, Stephanie Dellicour, Feiko Ter Kuile, Peter Ouma, Penny Phillips-Howard, Florence Were, Kayla Laserson and Meghna Desai [28]	BMC Pregnancy and Childbirth	2015	Eliminating fees associated with antenatal and delivery care is crucial. Addressing barriers requires investment in health promotion and transportation.
Childhood mortality in sub-Saharan Africa: cross-sectional insight into small-scale geographical inequalities from Census data	Lawrence Kazembe, Aileen Clarke, Ngianga-Bakwin Kandala	BMJ Open	2012	Most women preferred delivering in health facilities due to the better management of complications. Cost remained a barrier, leading some to visit TBAs. Addressing barriers requires investment in health promotion and transportation.
Lista de causas de muerte potencialmente evitables en la niñez: una propuesta para Colombia	Maylen Liseth Rojas-BoteroYadira Eugenia Borrero-RamírezFlor de María Cáceres-Manrique	Cadernos de Saúde Pública	2020	The study describes the geographical distribution of avoidable deaths (preventable and amenable to healthcare) in Colombia at the lower administrative level (civil parish). Considerable geographical inequalities exist between more advantaged and more deprived neighborhoods in Colombia. The identified causes of death were categorized as treatable (39.5%), preventables (47.4%), or mixed (13.1%). The study found consensus among experts in child health regarding the potential avoidability of these causes.
Child, maternal, and adult mortality in Sierra Leone: nationally representative mortality survey 2018–20	Ronald Carshon-Marsh, Ashley Aimone, Prof Rashid Ansumana, Ibrahim Bob Swaray, Anteneh Assalif Alimatu Musa, MSc et al.	The Lancet Global Health	2021	Malaria was the leading cause of death in children and adults, representing 22% of deaths under the age of 70 in 2020. Other infectious diseases accounted for an additional 16% of deaths in this age group. Sierra Leone has among the highest rates of maternal and neonatal mortality in the world. The maternal mortality ratio was 510 deaths per 100,000 live births, and the neonatal mortality rate was 31.1 deaths per 1000 live births. Hemorrhage was the major cause of maternal mortality, while birth asphyxia or trauma was the major cause of neonatal mortality. Excess deaths were not detected during the peak of the COVID-19 pandemic in Sierra Leone. Half of the deaths occurred in rural areas and at home. Excess deaths were not detected during the peak of the COVID-19 pandemic in Sierra Leone: Half of the deaths occurred in rural areas and at home.
Assessing the causes of under-five mortality and proportion associated with pneumococcal diseases in Cameroon. A case-finding retrospective observational study: 2006–2012	John Njuma Libwea, Sandrine Rachel Bebey Kingue, Nadesh Taku Ashukem, Marie Kobela, Angeline Boula, Koulla-Shiro Sinata, Paul Koki Ndombo	Randomized Controlled Trial	2019	Of the 817 death records assessed, malaria was the leading CoD and was responsible for 17.5% of cases. Meningitis was the second largest CoD with 11.0%; followed by sepsis (10.0%), Streptococcus pneumoniae infections (8.3%), malnutrition (8.3%), gastro-enteritis/diarrhea (6.2%), anemia (6.1%) and HIV (3.5%), respectively.
Determinants of infant mortality in community of Gilgel Gibe Field Research Center, Southwest Ethiopia: a matched case–control study	BMC Public Health	Lamessa Dube, Mohammed Taha, Henok Asefa	2013	Not attending the antenatal care follow-up was associated with an increased risk of infant mortality (Adjusted Odds Ratio [AOR] = 2.04, 95% Confidence Interval [CI]: 1.04, 4.02). Negative perceived benefits of mothers toward modern treatment and prevention were independently associated with infant mortality (AOR = 2.76, 95% CI: 1.21, 6.09). Small birth size (AOR = 2.91, 95% CI: 1.01, 8.46) and high birth order with short birth intervals (AOR = 3.80, 95% CI: 1.20, 11.98) were also significant determinants.
Access to paediatric surgery: the geography of inequality in Nigeria	Mohamed Abd Salam El Vilaly, Maureen A Jones, Makela Cordero Stankey, Justina Seyi-Olajide, Bisola Onajin-Obembe, Andat Dasogot, Stefanie J Klug, John Meara, Emmanuel A Ameh, Olabisi O Osagie, Sabrina Juran	BMJ Global Health	2021	69.5–98% of Nigeria’s 0–19 population lacks timely access to surgical care. Developing pediatric surgical services in underserved states and investing in training the pediatric surgical and anesthesia workforce are crucial steps to improve child health and reduce Nigeria’s burden of surgical disease.
Qualitative needs assessment for paediatric emergency care in Kampala, Uganda	Boyoung Ahn, Ezekiel Mupere, Belén Irarrázaval, Robert O Opoka, Hellen Aanyu-Tukamuhebwa, Corey B Bills, Dorothy Gingo, Nicolaus W Glomb	Africa Journal Emergency Medicine	2021	Of 35 pediatric assessment, treatment, and teamwork skills, 29 (83%) questions had the median comfort rating of 6 or 7 on a 7-point Likert scale. The remaining 6 (17%) skills had a median comfort rating of 5 or lower. Focus groups identified a number of major barriers to caring for critically ill children, including limited resources and staffing, training gaps, and challenges with interprofessional teamwork. In terms of training development, focus group participants suggested continuous training for all medical providers working in the PACU led by local leaders.
Trends and inequities in amenable mortality between 1997 and 2012 in South Africa	I Neethling, P Groenewald, H Schneider, D Bradshaw	South Africa Medical Journal	2019	The study found significant disparities in amenable mortality across provinces and population groups in South Africa. These disparities persisted over time and did not improve. While there was an average annual increase in amenable age-standardized death rates (ASDRs), excluding HIV/AIDS from the analysis showed an average annual decrease of 1.12%. During the post-peak HIV/AIDS period (2008–2012), an estimated 207,810 amenable deaths could have been prevented annually if all provinces had the same ASDR as the Western Cape. South Africa’s ASDR was 2.6 and 2.2 times higher than that of the worst-performing EU and OECD countries, respectively.
Trends in amenable mortality rate in the Mongolian population, 2007–2014	Enkhjin Surenjav, Tugsdelger Sovd, Yoshitoku Yoshida, Eiko Yamamoto, Joshua A. Reyer, and Nobuyuki Hamajima	Nagoya Journal of Medical Science	2016	Perinatal deaths and deaths due to influenza, pneumonia and asthma were highest in under-five children. There were great discrepancies in age-specific AMRs among provinces, especially in younger age groups.
Determinants of neonatal mortality in rural Northern Ethiopia: A population based nested case–control study	Robel Yirgu, Mitike Molla, Lynn Sibley	Plos One	2017	During the study period, the neonatal mortality rate was 18.6 (95% CI: 14.8, 23.2) per 1000 live births. Neonatal mortality declined with an increase in family size. Neonates born into families with more than two members had lower odds of death in the neonatal period compared to those born into families of two members (Adjusted Odds Ratio [AOR] = 0.13, 95% CI: 0.02, 0.71). Mothers who gave birth to 2–4 children had lower odds of losing their newborns to neonatal mortality (AOR = 0.15, 95% CI: 0.05, 0.48). Mothers who gave birth to 5 or more children also had lower odds of neonatal mortality (AOR = 0.08, 95% CI: 0.02, 0.26).
Understanding the Rapid Increase in Life Expectancy in South Korea	Seungmi Yang, Young-Ho Khang, George Davey Smith, David A. Leon, and John Lynch	American Journal of Public Health	2010	Infectious diseases, such as pneumonia and influenza, were historically major contributors to child mortality. However, effective prevention strategies and medical advancements have reduced their impact.
Avoidable, amenable, and preventable mortalities in South Korea, 2000–2017: Age-period-cohort trends and impact on life expectancy at birth	Sang Jun Eun	Social Science and Medine	2019	Avoidable, treatable, and preventable mortalities in Korea declined at different rates over time by region. Absolute and relative regional disparities in avoidable and preventable mortalities generally decreased. However, relative disparities in treatable mortality between areas widened. After around 2009, regional disparities in all types of mortalities tended to improve, especially among males. In females, disparities in avoidable, treatable, and preventable mortalities between areas improved less or even worsened.
Origins of disparities in preventable child mortality in England and Sweden: a birth cohort study	Ania Zylbersztejn, Ruth Gilbert, Anders Hjern, Pia Hardelid	Arch Dis Child Archives of Disease in Chilhood	2020	At 31–364 days, the unadjusted hazard ratio (HR) for RTI-related death in England versus Sweden was 1.52. After adjusting for birth characteristics and socioeconomic factors, the HR reduced to 1.11.
Socioeconomic position, health behaviors, and racial disparities in cause-specific infant mortality in Michigan, USA	Abdulrahman M. El-Sayed, Darryl W. Finkton, Jr, Magdalena Paczkowski, Katherine M. Keyes, and Sandro Galeaa	Preventive Medicine	2015	Racial differences in SEP and maternal risk behaviors explain some, but not all, excess infant mortality among blacks relative to non-Hispanic whites. These factors contribute to disparities in specific causes of infant mortality.
Mortality in children admitted to Port Moresby General Hospital: how can we improve our hospital outcomes?	Titus Nasi, John D Vince, David Mokela	Papua and Nueva Guinea Medical Journal	2003	238 children died out of the 4898 admitted, resulting in an overall case fatality rate of 4.9%. The proportion of deaths roughly matched the proportion of admissions in each age group: 92% of the deceased children had a weight of less than 80% of the standard weight for their age; 30% weighed less than 60% of the standard weight for their age. The four leading certified causes of death were: pneumonia, meningitis, measles, septicemia
Associations Between Race/Ethnicity and US Childhood and Adolescent Cancer Survival by Treatment Amenability	Arash Delavar, Justin M. Barnes, Xiaoyan Wang, and Kimberly J. Johnson	JAMA Pediatrics	2020	Racial and ethnic minority children and adolescents with cancer had worse survival compared to non-Hispanic white children and adolescents. The disparity was generally greater for cancer types with higher relative survival rates (RSRs).
Rates and causes of mortality among children and young people with and without intellectual disabilities in Scotland: a record linkage cohort study of 796 190 school children	Gillian S Smith, Michael Fleming, Deborah Kinnear, Angela Henderson, J P Pell, Craig Melville, Sally Ann Cooper	BMJ open	2020	The most common main underlying causes of death were diseases of the nervous system, followed by congenital anomalies. The most common all-contributing causes included diseases of the nervous system, followed by the respiratory system. The most common specific contributing causes were cerebral palsy, pneumonia, respiratory failure, and epilepsy. External causes accounted for 46% of control deaths, but the SMR for external-related deaths was still higher (3.6, 95% CI: 2.2 to 5.8) for students with intellectual disabilities.
Association between enrolment with a Primary Health Care provider and amenable mortality: A national population-based analysis in Aotearoa New Zealand	Pushkar Silwal, Maite Irurzun Lopez, Megan Pledger, Jacqueline Cumming, Mona Jeffreys	Plos One	2023	106 children died out of 67,342 person-years, resulting in a crude mortality rate of 157/100,000 person-years. Being enrolled in a PHC system is associated with a lower level of amenable mortality.
Contribution of amenable mortality to life expectancy differences between the Dutch Caribbean islands of Aruba and Curaçao and the Netherlands	Soraya P. A. Verstraeten, Hans A. M. van Oers, and Johan P. Mackenbach	Pan American Journal of Public Health	2020	106 children died over 67,342 person-years in Aruba and Curaçao, resulting in a crude mortality rate of 157/100,000 person-years. In comparison, 458 controls died over 3,672,224 person-years in the Netherlands, with a crude mortality rate of 12/100,000 person-years. The Age–SMR was 11.6 (95% CI: 9.6 to 14.0) for female pupils and 9.8 (95% CI: 7.7 to 12.5) for male pupils. The largest cause-specific contributions were found for: circulatory diseases, breast cancer, perinatal causes, nephritis/nephrosis (solely in Curaçao)
Community surveillance and response to maternal and child deaths in low- and middle-income countries: A scoping review	Tariro J. Basera et al.	Plos One	2021	Least Severe Scenario (6 Months): Coverage reductions of 9.8–18.5% and a wasting increase of 10%. Result: 253,500 additional child deaths and 12,200 additional maternal deaths. Most Severe Scenario (6 Months): Coverage reductions of 39.3–51.9% and a wasting increase of 50%. Result: 1,157,000 additional child deaths and 56,700 additional maternal deaths.
Risk factors for mortality among neonates admitted to a special care unit in a low-resource setting	Francesco Cavallin, Teresa Bonasia, Desalegn Abebe Yimer, Fabio Manenti, Giovanni Putoto and Daniele Trevisanuto	BMC Pregnancy and Childbirth	2020	Risk Factors Associated with Increased Mortality: Neonates referred from other health facilities or home (odds ratio 1.52). Moderate hypothermia at admission (odds ratio 1.53). Diagnosis of late-onset sepsis (odds ratio 1.63). Low birthweight (odds ratio 2.48). Very low birthweight (odds ratio 11.71). Extremely low birthweight (odds ratio 76.04). Intrapartum-related complications (odds ratio 4.69). Meconium aspiration syndrome (odds ratio 2.34). Respiratory distress (odds ratio 2.25). Other infections (odds ratio 1.92). Malformations (odds ratio 2.32). Factors Associated with Decreased Mortality: Being admitted in 2017 vs. 2014 (odds ratio 0.71). Older age at admission (odds ratio 0.95).
Neonatal Transport in the Practice of the Crews of the Polish Medical Air Rescue: A Retrospective Analysis	Ewa Rzońca, Stanisław Paweł Świeżewski, Robert Gałązkowski, Agnieszka Bień, Arkadiusz Kosowski, Piotr Leszczyński, and Patryk Rzońca	International Journal of Environmental Research of Public Health	2020	Risk Factors Associated with Increased Mortality: Neonates referred from other health facilities or home. Moderate hypothermia at admission. Diagnosis of late-onset sepsis. Low birthweight, very low birthweight, and extremely low birthweight. Intrapartum-related complications, meconium aspiration syndrome, respiratory distress, other infections, and malformations.
Avoidable mortality among parents whose children were placed in care in Sweden: A retrospective matched cohort study	Elizabeth Wall-Wieler Bo Vinnerljung Can Liu Leslie Roos Anders Hjern	International Journal of Population Data Science	2018	In comparison to parents who did not have a child placed in care, those with children placed in care experienced additional avoidable deaths. Mothers: An additional 21 avoidable deaths per 10,000 person-years. Death due to preventable causes was 3.83 times greater (95% CI 2.82–5.21). Deaths due to amenable causes were 3.12 times greater (95% CI 2.07–4.69). Higher avoidable mortality rates for mothers whose children were young when placed in care. Fathers: An additional 27 avoidable deaths per 10,000 person-years. Death due to preventable causes was 1.75 times greater (95% CI 1.41–2.16). Deaths due to amenable causes were 1.52 times greater (95% CI 1.08–2.13). Parents whose children were all placed in care also had higher avoidable mortality rates
Does child gender determine household decision for health care in rural Thatta, Pakistan?	R Nuruddin, W C Hadden, M R Petersen, M K Lim	Journal of Public Health (Oxford)	2009	There were 25 more girl deaths than boys per 1000 live births among post-neonates and 38 more among children aged 12–59 months. However, in an adjusted analysis, gender was not a significant predictor of illness reporting, visits to health facilities choice of health care provider, hospitalization, or health expenditure.
Mortality among children in Basrah	Omran S Habib, Suham A Warid	The Medical Journal Of Basrah University	2015	Neonatal mortality rate (NMR): 16.7 per 1000 live births. Post-neonatal mortality rate (PNMR): 4.6 per 1000 live births. Child mortality rates (1–4 years): 3.1 per 1000 live births. Infant mortality rate (IMR): 21.3 per 1000 live births. The highest mortality rates were recorded in the Al-Hartha and Shat-Al-Arab districts. The Al-Fao district showed the highest PNMR.
Inequalities in child mortality in ten major African cities	Wilm Quentin, Olayinka Abosede, Joseph Aka, Patricia Akweongo, Kouassi Dinard, Alex Ezeh, Ramadan Hamed, Patrick Kalambayi Kayembe, Getnet Mitike, Gemini Mtei, Marguerite Te Bonle and Leonie Sundmacher	BMC medicine	2014	Significant inequalities were observed in several cities, including Kinshasa, Luanda, Abidjan, and Addis Ababa.
Understanding rapid implementation from discovery to scale: Rwanda’s implementation of rotavirus vaccines and PMTCT in the quest to reduce under-5 mortality	Felix Sayinzoga, Lisa R. Hirschhorn, Jovial Thomas Ntawukuriryayo, Caroline Beyer, Kateri B. Donahoe and Agnes Binagwaho	BMC pediatrics	2024	Rwanda was the first low-income African country to implement the rotavirus vaccine (RTV) and adopt Option B+ for the effective prevention of mother-to-child transmission (PMTCT) before the World Health Organization’s (WHO) recommendation.
Lessons from Peru to reduce under-5 mortality: understanding program implementation and context	Patricia J. García, Anna Larson Williams, Marco H. Carcamo, Amelia VanderZanden and Agnes Binagwaho	BMC Pediatrics	2024	The percentage of mothers attending at least four antenatal care visits increased from 69% to 96.9%, and facility-based deliveries rose from 56% to 91%. Additionally, tetanus/diphtheria/pertussis vaccine coverage reached 90% by 2015. Key factors contributing to this success included economic growth, financial reforms, a national commitment to poverty reduction, and the prioritization of maternal and child health. Strategies such as data-driven decision-making, cultural adaptation, and equity-focused initiatives played a crucial role in achieving these positive outcomes
A comparison of child mortality from potentially preventable causes in England and Sweden using birth cohorts from linked administrative datasets	Ania Zylbersztejn Ruth Gilbert Anders Hjern Pia Hardelid	Conference Proceedings for International Population Data Linkage Conference 2018	2018	For RTI-related deaths at 31–364 days: Unadjusted HR (England vs. Sweden): 1.50. Adjusted for birth characteristics: 1.16. Adjusted for socioeconomic factors: 1.11. For RTI-related deaths at 1–4 years: Unadjusted HR (England vs. Sweden): 1.58. Adjusted for birth characteristics: 1.32. Adjusted for socioeconomic factors: 1.30. For SUDIs at 31–364 days: Unadjusted HR (England vs. Sweden): 1.59. Adjusted for birth characteristics: 1.40. Adjusted for socioeconomic factors: 1.19.
Mortality of American Indian and Alaska native infants	E R Rhoades, G Brenneman, J Lyle, A Handler	Annual Review Public Health	1992	American Indian and Alaska Native infants face significant disparities in mortality rates compared to non-Hispanic white infants. Nationwide, AI/AN infants are nearly twice as likely to die by their first birthday as non-Hispanic white infants. The most common causes of infant mortality among AI/AN infants include: congenital malformations (birth defects), sudden infant death syndrome (SIDS), and prematurity.
Why do UK-born Pakistani babies have high perinatal and neonatal mortality rates?	S Bundey, H Alam, A Kaur, S Mir, R Lancashire	Paediatric Perinatal Epidemiology	1991	UK-born Pakistani babies experience a high perinatal mortality rate compared to other ethnic groups. The major cause of early mortality in UK-born Pakistani babies is a high rate of lethal malformations. These malformations occur in about 1 in 100 Pakistani babies and account for approximately half of their perinatal mortality. Many of these malformations are autosomal recessive and occur only in offspring of consanguineous parents. In addition to lethal malformations, there is also an excess of lethal cardiac malformations among UK-born Pakistani babies.
Estimated impact of the 2020 economic downturn on under-5 mortality for 129 countries	Marcelo Cardona, Joseph Millward, Alison Gemmill, Katelyn Jison Yoo, David M Bishai	Plos one	2022	A 5% reduction in GDP per capita in 2020 was estimated to cause an additional 282,996 deaths in children under 5 in 2020. At 10% and 15%, recessions led to higher losses of under-5 lives, increasing to 585,802 and 911,026 additional deaths, respectively.
Household factors and under-five mortality in Bankass, Mali: results from a cross-sectional survey	David C. Boettiger, Emily Treleaven, Kassoum Kayentao, Mahamadou Guindo, Mama Coumaré, Ari D. Johnson, Caroline Whidden, Naimatou Koné, Amadou Beydi Cissé, Nancy Padian and Jenny Liu	BMC Public Health	2021	Distance from healthcare: Living a greater distance from healthcare facilities is associated with increased U5 mortality.
Determinants of infant mortality in community of Gilgel Gibe Field Research Center, Southwest Ethiopia: a matched case–control study	Lamessa Dube, Mohammed Taha and Henok Asefa	BMC Public Health	2013	Antenatal care, hand washing habits, birth size, maternal perception of modern treatments, and birth intervals were determinants of infant mortality in this community

## Data Availability

Not applicable.
